# Oral midodrine for prophylaxis against post-spinal anesthesia hypotension during hip arthroplasty in elderly population: a randomized controlled trial

**DOI:** 10.1186/s12871-024-02442-8

**Published:** 2024-02-14

**Authors:** Sarah Amin, Ahmed Hasanin, Rehab Mansour, Maha Mostafa, Dina Zakaria, Amany S Arafa, Akram Yassin, Hisham Ziada

**Affiliations:** Department of Anesthesia and Critical Care Medicine, Faculty of Medicine, Cairo University, Cairo, Egypt

**Keywords:** Spinal anesthesia, Hypotension, Hip arthroplasty, Elderly, Midodrine, Ephedrine

## Abstract

**Background:**

We aimed to evaluate the efficacy of midodrine as a prophylaxis against post-spinal hypotension in elderly patients undergoing hip arthroplasty.

**Methods:**

This randomized controlled trial included elderly patients undergoing hip arthroplasty under spinal anesthesia. Ninety minutes before the procedure, patients were randomized to receive either 5-mg midodrine or placebo (metoclopramide). After spinal anesthesia, mean arterial pressure (MAP) and heart rate were monitored every 2 min for 20 min then every 5 min until the end of the procedure. Post-spinal hypotension (MAP < 80% baseline) was treated with 10 mg ephedrine. The primary outcome was intraoperative ephedrine consumption. Secondary outcomes were the incidence of post-spinal hypotension, bradycardia, and hypertension (MAP increased by > 20% of the baseline reading).

**Results:**

We analyzed 29 patients in the midodrine group and 27 in the control group. The intraoperative ephedrine consumption was lower in the midodrine group than in the control group (median [quartiles]: 10 [0, 30] mg versus 30 [20, 43] mg, respectively, *P*-value: 0.002); and the incidence of intraoperative hypotension was lower in the midodrine group than that in the control group. The incidence of hypertension and bradycardia were comparable between the two groups.

**Conclusion:**

The use of 5 mg oral midodrine decreased the vasopressor requirements and incidence of hypotension after spinal anesthesia for hip surgery in elderly patients.

**Clinical trial registration:**

This study was registered on September 22, 2022 at clinicaltrials.gov registry, NCT05548985, URL: https://classic.clinicaltrials.gov/ct2/show/NCT05548985.

## Introduction

Hip arthroplasty is performed for various underlying pathologies and most patients undergoing this procedure are elderly [1]. Neuraxial anesthesia is the preferred route of anesthesia since it reduces the risk of postoperative complications [2, 3]. However, elderly patients are at increased risk of post-spinal anesthesia hypotension [4]. Intraoperative hypotension compromises vital organ perfusion which consequently increases the risk of kidney injury, brain ischemia, cardiac ischemia, and 30-day mortality [5, 6]. Elderly patients commonly have systemic medical disorder; therefore, they are highly vulnerable to the complications of hypotension [7]. Thus, intraoperative hypotension during hip surgery has been recently recognized as a major risk factor for postoperative morbidity and mortality [8].

Through the inhibitory effect of spinal anesthesia on the sympathetic nervous system, hypotension is induced due to vasodilatation which decreases the venous return, and consequently the cardiac output [9]. Therefore, the main line for prevention and management of spinal anesthesia- induced hypotension is vasopressors [10]. The commonly used vasopressor drugs in the perioperative settings are alpha-adrenoreceptor agonists such as ephedrine, phenylephrine, and recently norepinephrine. All these agents are effective in maintaining blood pressure; however, they have some disadvantages; ephedrine is commonly associated with tachycardia (due to the beta-agonist activity) [11], phenylephrine and norepinephrine are usually associated with reactive bradycardia [12]. Furthermore, all the mentioned drugs are used intravenously.

Midodrine hydrochloride is another alpha-adrenoreceptor agonist which has the advantage of being an oral drug with minimal central nervous system side effects, and good oral bioavailability [13]. Midodrine is metabolized to desglymidodrine which is a direct arteriolar and venous vasopressor [14]. Midodrine is used for management of some hypotensive disorders. Midodrine was previously used for management of orthostatic hypotension [13], intra-dialysis hypotension [15], and in circulatory shock in critically ill patients [16]. There is paucity of data about the value of midodrine in preoperative settings. in this study we aimed to evaluate the efficacy of midodrine as a prophylaxis against post-spinal hypotension in elderly patients undergoing hip arthroplasty.

## Patients and methods

This randomized controlled trial was conducted at Cairo University Hospital between October 2022 to July 2023, after Cairo University Research Ethics Committee approval (MD-25-2021). Trial registration was done prior to patients’ enrollment at clinicaltrials.gov (NCT05548985, date: 22/09/2022). Written informed consent was obtained from the patient before the enrolment.

Participants were elderly patients (> 65 years), American society of anesthesiologists (ASA)-physical status I-III, scheduled for hip arthroplasty under spinal anesthesia.

Exclusion criteria were patients with hypertension, impaired cardiac contractility, significant stenotic lesions, chronic beta-blocker or digoxin therapy, significant arrhythmia, history of glaucoma, liver cell failure, renal impairment, and contraindications of spinal anesthesia.

Randomization sequence was obtained via an online randomization site in a 1:1 ratio. The randomization number, group assignment and drug preparation instruction were enclosed in opaque sequentially numbered envelopes. An independent research assistant handled envelope opening and drug preparation. The assigned drug was given to the attending nurse. Neither the research assistant nor the attending nurse had further involvement in the study.

### Preoperative management

Participants were instructed to fast for 6 h for solid light meals and 2 h for clear fluids. In the preparation, standard monitors (noninvasive blood pressure, pulse oximetry, 5-lead electrocardiogram) were applied and intravenous access was obtained. Baseline blood pressure was recorded as the average of three consecutive measurements with variability less than 10% in mean arterial pressure.

Inferior vena cava (IVC) collapsibility ([*maximum IVC diameter - minimum IVC diameter] / maximum IVC diameter)* was used to assess the patient’s intravascular volume status. Patients were considered responders when the IVC collapsibility > 36% and received 6 mL/kg Ringer’s lactate solution over 15 min [17]. The IVC examination was performed using low frequency convex transducer (2-5 MHz) connected to ACUSON Freestyle (Siemens Medical Solutions, Inc. USA).

Ninety min before the spinal anesthesia, patients received the study drug according to the randomization. Patients in the midodrine group received oral midodrine tablet (Midotab 5 mg, DRUG OCEAN pharmaceuticals, NJ, USA). Patients in the Control group received oral placebo (metoclopramide 10 mg). Metoclopramide tablet (Primperan 10 mg, Sanofi Egypt, Cairo, Egypt) was chosen as a placebo because it has the same appearance as midodrine tablet with no cardiovascular effects. Patients were monitored for blood pressure and heart rate at 15 min intervals after administration of the study drug.

### Anesthetic management

Spinal anesthesia was achieved by injecting 10-12.5 mg of 0.5% hyperbaric bupivacaine plus 25 mcg fentanyl into the subarachnoid space. The degree of sensory block (cold test by alcohol gauze) was assessed with a goal of at least T6-8 dermatomal level block. If spinal anesthesia failed, the patient was excluded from the study and was managed according to the attending anesthetist discretion. After induction of spinal anesthesia 2 mL/kg/hr of Ringer lactate were administered. After spinal anesthesia, mean arterial pressure and heart rate were monitored every 2 min for 20 min then every 5 min till the end of the procedure.

Post spinal hypotension was defined as mean arterial pressure < 80% of the baseline reading up to 45 min after spinal block. Hypotension was managed by 10 mg ephedrine which was repeated if hypotension persisted > 2 min. Bradycardia (heart rate < 50 bpm) was managed with 0.5 mg of atropine intravenous.

Further fluid and hemodynamic management were according to the discretion of the attending anesthetist.

The study’s primary outcome was intraoperative ephedrine consumption. The secondary outcomes were the incidence of post-spinal hypotension, bradycardia, and hypertension (defined as increased mean arterial pressure by > 20% of the baseline reading), mean arterial pressure and heart rate (at the prespecified time points).

### Sample size

In a pilot study on 14 elderly patients (seven in each group), the intraoperative ephedrine consumption was 17 ± 15 mg in the midodrine group and was 33 ± 24 mg in the control group. A minimum sample of 52 patients (26 patients in each group) would achieve 80% power to reject the null hypothesis of equal means between the two groups, and with a significance level (alpha) of 0.05 using a two-sided two-sample unequal-variance t-test. To compensate for any dropouts, the final number of envelopes was 58 (29 per group). sample size was calculated using PASS 15 Power Analysis and Sample Size Software (2017) (NCSS, LLC. Kaysville, Utah, USA).

### Statistical analysis

Analysis of data was performed using Statistical package for social science (SPSS) software, version 26 for Microsoft Windows (IBM Crop., NY, USA). Categorical data are reported as numbers and percentages and were analyzed using the Chi-square test. Normality of the continuous data were checked using the Kolmogorov-Smirnov test. Normally distributed data are presented as means ± standard deviations and were analyzed using the unpaired Student t-test. Skewed data are presented as medians (quartiles) and were analyzed using the Mann Whitney U test. For repeated measured data (heart rate and mean arterial pressure), analysis of variance for repeated measure was used to evaluate drug (between-groups factor) and time (repeated measures) effect. Adjustment for multiple comparison was achieved by the Bonferroni test. *P* value < 0.05 was considered significant.

## Results

**Fig. 1:**
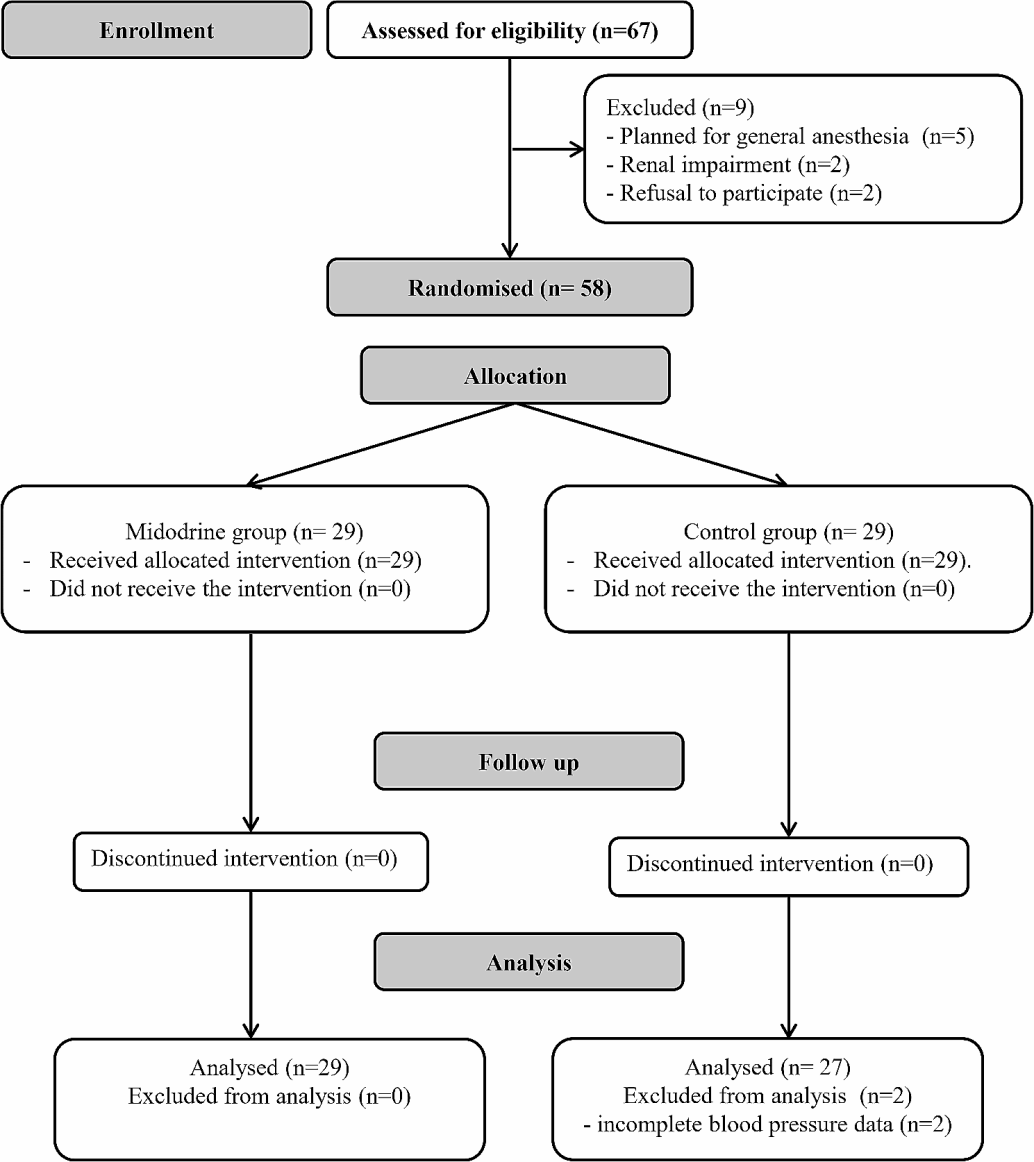
Consort’s flow chart

Sixty-seven patients were screened for eligibility, nine patients were excluded for not fulfilling the inclusion criteria. Fifty-eight patients were included (29 patients in each group) and were randomized to the study’s groups. Two patients in the control group were excluded for incomplete data and 56 patients were analyzed (29 in the midodrine group and 27 in the control group). (Fig. 1)

Baseline demographic data and hemodynamic characteristics were comparable between the two groups. (Table 1)

**Table 1:** Demographic data and baseline hemodynamic characteristics. Data presented as mean ±standard deviation, median (quartiles), and frequency (%)

	Midodrine group (*n* = 29)	Control group (*n* = 27)	*P*-value
Age (years)	78 (72, 85)	79 (75, 85)	0.532
Male sex	19 (66%)	17 (63%)	0.359
Weight (kg)	78 ± 9	77 ± 8	0.634
Baseline heart rate (bpm)	78 ± 12	76 ± 12	0.516
Baseline mean arterial pressure (mmHg)	88 ± 12	91 ± 10	0.259
Fluid responder	23 (79%)	24 (89%)	0.329

The intraoperative ephedrine consumption was lower in the midodrine group than in the control group (median [quartiles]: 10 [0, 30] mg versus 30 [20, 43] mg, respectively, *P*-value: 0.002); and the incidence of intraoperative hypotension was lower in the midodrine group than in the control group (15 [52%] versus 25 [93%] respectively, *P*-value: 0.001). (Table 2)

**Table 2:** Intraoperative outcomes. Data presented as median (quartiles), and frequency (%)

	Midodrine group (*n* = 29)	Control group (*n* = 27)	*P*-value
Ephedrine consumption (mg)	10 (0, 30)	30 (20, 43)	0.002
Incidence of hypotension	15 (52%)	25 (93%)	0.001
Incidence of hypertension	3 (10%)	2 (7%)	0.700
Incidence of bradycardia	5 (17%)	1 (4%)	0.102

**Fig. 2:**
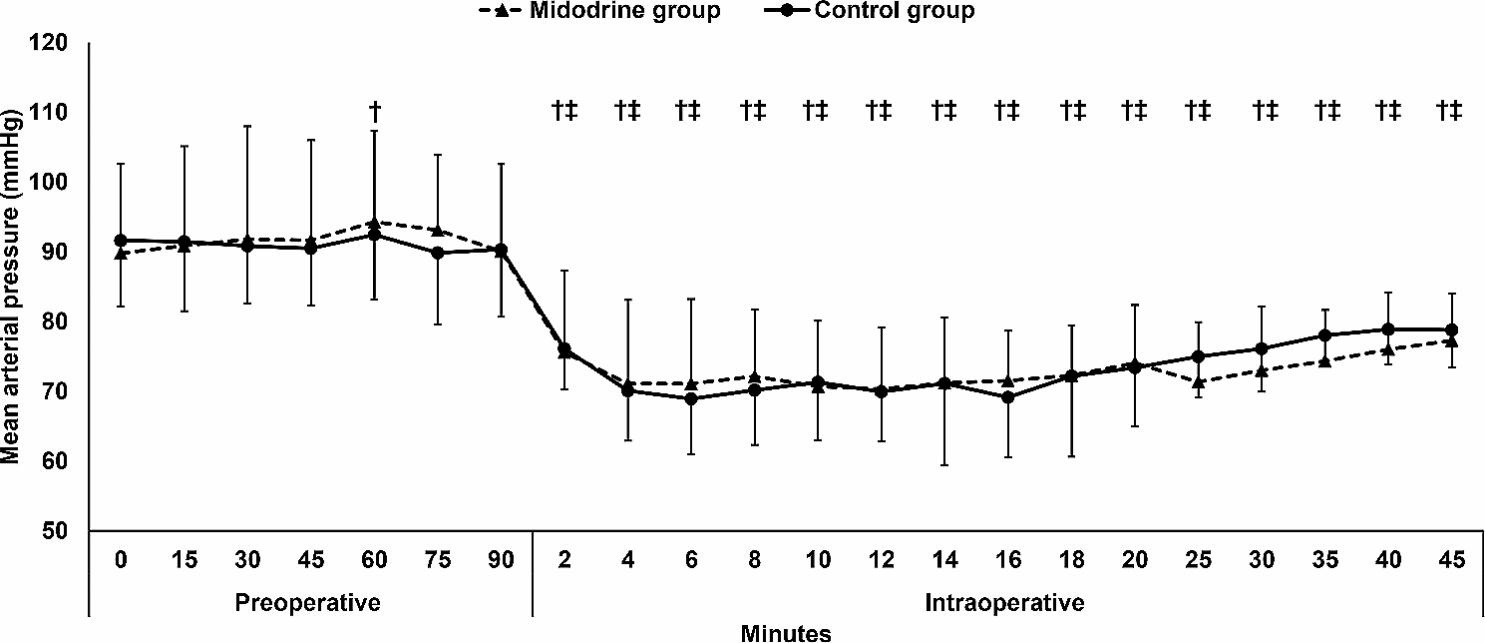
Mean arterial pressure. Markers are means and error bars are their standard deviations. † denotes significance in relation to baseline value in the Midodrine group, ‡ denotes significance in relation to baseline value in the control group

**Fig. 3:**
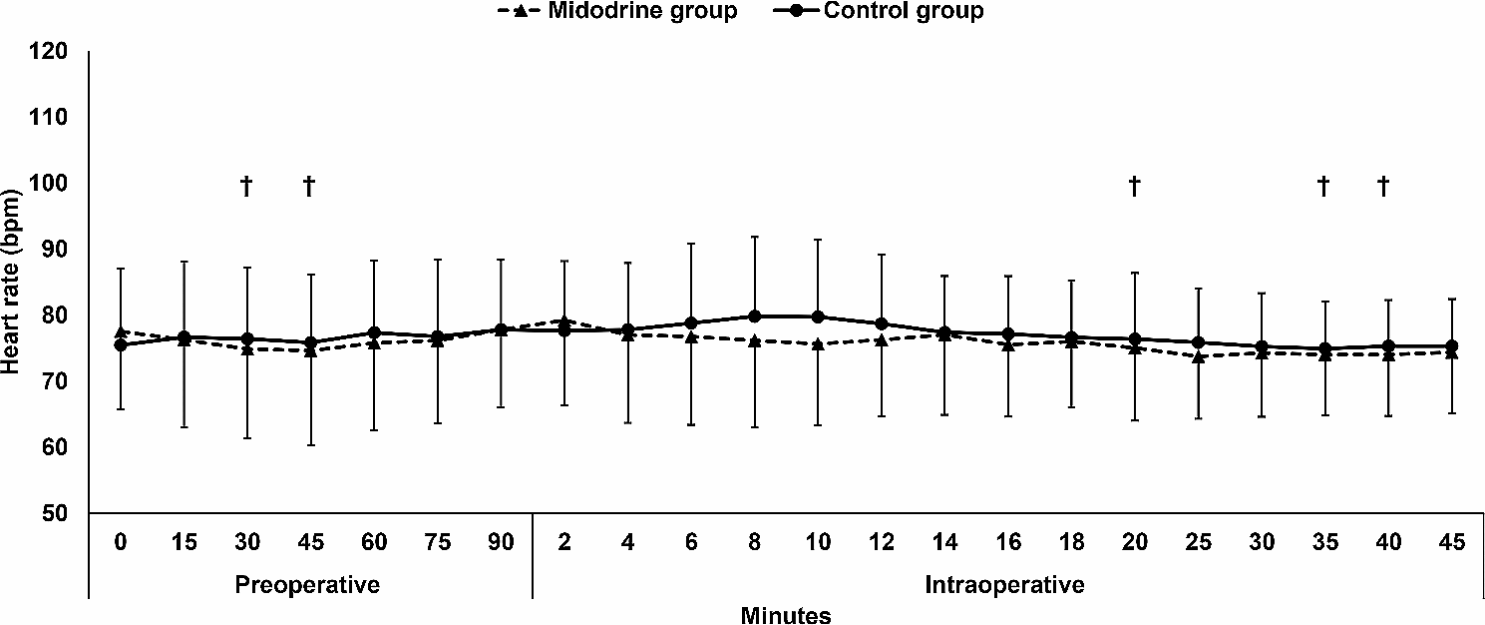
Heart rate. Markers are means and error bars are their standard deviations. † denotes significance in relation to baseline value in the Midodrine group

The incidence of hypertension and bradycardia were comparable between the two groups. (Table 2) Furthermore, the mean arterial pressure and heart rate were comparable between the two groups. (Figures 2 and 3)

In both groups, the intraoperative mean arterial pressure decreased in relation to the baseline reading, while the heart rate was generally maintained. (Figures 2 and 3)

## Discussion

We evaluated the use of preoperative oral midodrine in elderly population before spinal block and found that the intervention improved the intraoperative hemodynamic profile decreasing the vasopressor consumption and the incidence of hypotension denoting better maintenance of the blood pressure compared to control placebo.

Midodrine is an oral α1-adrenoreceptor agonist. Midodrine is a prodrug which is metabolized to desglymidodrine; the later molecule produces direct arteriolar and venous vasoconstriction and, consequently, increases the systemic vascular resistance [18]; these pharmacologic properties explain the vasopressor-sparing effect of midodrine in our patients. Several studies in different populations support the positive cardiovascular effects of midodrine. The most established effect for midodrine is the treatment of chronic orthostatic hypotension [13]. Midodrine also improved the symptoms of dialysis-induced hypotension [15]. On the other hand, midodrine showed controversial results in some groups of patients such as critically ill patients with circulatory shock and postoperative surgical patients [16, 19]. The inconsistency in the results of the drug among different populations highlights the importance of adequate evaluation in various clinical settings before reaching a recommendation for its use.

The use of midodrine for prophylaxis against post-spinal hypotension had not been adequately evaluated. A randomized controlled trial showed that the drug can decrease the incidence of post-spinal hypotension in adult non-elderly population [20]. Our study is the first to evaluate the use of midodrine in the special subgroup of elderly patients. Intraoperative hypotension is a serious independent risk factor perioperative mortality in patients undergoing hip surgery [21]. Spinal anesthesia is the usual choice for anesthesia in hip surgery and is commonly associated with hypotension which has higher prevalence in elderly compared to young adults. Furthermore, elderly patients are more vulnerable to complications of hypotension due to age-related cardiovascular changes such as increased basal sympathetic activity and reduced baroreceptor sensitivity [22]. It was found that pre-hydration alone would not eliminate spinal hypotension [23, 24]. Therefore, there is increased interest in the use of vasopressors, preferably prophylactic, to decrease the incidence and severity of hypotension [25].

We used a dose of 5 mg of the drug administered 90 min before spinal anesthesia. The usual dose of the drug in previous studies ranged between 2.5- and 10 mg per dose [13, 15, 19]. The peak effect of desglymidodrine ranges between 60 and 120 min after oral administration of midodrine [14]. The effects of other doses and time of drug administration require further investigation.

According to our results, we suggest that the use of midodrine would maintain stable blood pressure in elderly patients receiving spinal block. We chose the total vasopressor requirements rather than the incidence of hypotension as primary outcome because the former reflects both the frequency and severity of hypotension.

Our study has several important advantages such as the randomized controlled design and adequate sample size. Furthermore, the median age in our patients 78 years which represents a highly vulnerable group of elderly patients. There are some limitations which should be addressed in the study such as being performed in single center, the use of one dose of the drug, and excluding patients with significant cardiovascular pathologies. These limitations warrant future appropriate studies.

In conclusion, the use of 5 mg oral midodrine decreased the vasopressor requirements and incidence of hypotension after spinal anesthesia for hip surgery in elderly patients.

## Data Availability

The datasets used and/or analyzed during the current study are available from the corresponding author on reasonable request.
